# Recent Progress in Machine Learning-Based Methods for Protein Fold Recognition

**DOI:** 10.3390/ijms17122118

**Published:** 2016-12-16

**Authors:** Leyi Wei, Quan Zou

**Affiliations:** School of Computer Science and Technology, Tianjin University, Tianjin 300354, China; weileyi@tju.edu.cn

**Keywords:** protein fold recognition, machine learning, computational method

## Abstract

Knowledge on protein folding has a profound impact on understanding the heterogeneity and molecular function of proteins, further facilitating drug design. Predicting the 3D structure (fold) of a protein is a key problem in molecular biology. Determination of the fold of a protein mainly relies on molecular experimental methods. With the development of next-generation sequencing techniques, the discovery of new protein sequences has been rapidly increasing. With such a great number of proteins, the use of experimental techniques to determine protein folding is extremely difficult because these techniques are time consuming and expensive. Thus, developing computational prediction methods that can automatically, rapidly, and accurately classify unknown protein sequences into specific fold categories is urgently needed. Computational recognition of protein folds has been a recent research hotspot in bioinformatics and computational biology. Many computational efforts have been made, generating a variety of computational prediction methods. In this review, we conduct a comprehensive survey of recent computational methods, especially machine learning-based methods, for protein fold recognition. This review is anticipated to assist researchers in their pursuit to systematically understand the computational recognition of protein folds.

## 1. Introduction

Understanding how proteins adopt their 3D structure remains one of the greatest challenges in science. Elucidation of this process would greatly impact various fields of biology and medicine, as well as the rational design of new functional proteins and drug molecules. Determination of the fold category of a protein is crucial as it reveals the 3D structure of proteins. Classification of a protein of unknown structure under a fold category is called fold recognition, which is a fundamental step in the determination of the tertiary structure of a protein.

In the early years, determination of protein structure relies on traditional experimental methods, such as X-ray crystallography and nuclear magnetic resonance spectroscopy. In the post-genomic era, numerous sequences are generated by next-generation sequencing techniques. Although an increasing number of sequences are structurally characterized using experimental methods, the gap between structurally determined sequences and uncharacterized sequences is constantly increasing. Therefore, developing computational methods for fast and accurate determination of protein structures is urgently needed. Accurate computational prediction of protein folds has recently emerged as alternative approach to the labor intensive and expensive experimental methods. Computational methods for protein fold recognition can be generally categorized into three classes: (1) de novo modeling methods; (2) template-based methods; and (3) template-free methods. Many efforts have focused on the development of methods under classes (2) and (3) because the de novo approach (class 1) has two limitations. First, it requires long computational time and numerous sources, and second, it can only be successfully applied in small proteins.

Template-based methods used to determine protein structures are based on the evolutionary relationships of proteins. The procedure for template-based methods can be summarized as follows: First, proteins of known structures retrieved from public protein structure databases (e.g., Protein Data Bank (PDB)) are used as template proteins for a query protein sequence. To make template-based prediction fast and reliable, a simplified database is usually employed, in which the sequence similarity is less than 50%–70%. Second, distant evolutionary relationships between a target sequence and proteins of known structure are detected. In this step, multi-alignment algorithms are adopted to exploit evolutionary information by encoding amino acid sequences into profiles. Third, to determine the optimal alignments, scoring functions are usually used as measures to evaluate the similarity between the profiles derived from a query protein and those of template proteins with known structures. *Z*-score and *E*-value are the two commonly used scoring functions. The accuracy of the alignment is tremendously important in model building. Fourth, 3D structure models based on template atom coordinates and optimal query-template alignments are built. Last, the optimal structure models are determined from the model candidates through further structure optimization. The commonly used structural optimization methods include energy minimization and loop modeling.

A series of template-based methods were developed in the last few decades. This series of approaches are regarded as the most successful methods in constructing theoretical models of protein structures. For instance, Jaroszewski et al. [1] developed a protein recognition method called Fold and Function Assignment System (FFAS) by using a profile-profile alignment strategy without using any structural information. In FFAS, query and template profiles are obtained by PSI-BLAST searching against the NR85 database; these profiles are then aligned by a dot-product scoring function. The significance of alignment scores was calculated by comparing the protein with the distribution scores from pairs of unrelated proteins. Xu et al. [2] improved the FFAS method and proposed a method called FFAS-3D, wherein they introduced structural information, such as secondary structure, solvent accessibility, and residue depth. FFAS-3D remarkably outperforms FFAS. Moreover, Shi et al. [3] developed a protein fold recognition method called FUGUE, which can search sequences against protein fold libraries by using environment-specific substitution tables and structure-dependent gap penalties. Raptor is a novel method that uses the mathematical theory of linear programming to build 3D models of proteins and predict protein folds [4,5]. Roy et al. [6] developed an online prediction server called I-TASSER (Iterative Threading ASSEmbly Refinement), which is an integrated platform for automated protein structure and function prediction based on the sequence-to-structure-to-function paradigm. Ghouzam et al. [7] proposed ORION, a new fold recognition method based on pairwise comparison of hybrid profiles that contain evolutionary information from protein sequences and their structures. Other template-based methods were successfully developed, including MODELLER [8] and TMFR [9]. MODELLER implements comparative protein structure modeling through satisfaction of spatial restraints, whereas TMFR applies special scoring functions to align sequences and predict whether given sequence pairs share the same fold. As mentioned above, several typical template-based methods have been proposed. However, the manner by which to examine the quality of template-based modeling methods remains unknown. Currently, CASP (Critical Assessment of protein Structure Prediction) is a mainstream platform used to establish an independent mechanism to assess the current methods employed in protein structure modeling [10]. This platform can be accessed at http://predictioncenter.org/.

Although much progress has been made in template-based methods, some problems still exist, as follows: First, we need to determine the structures of template proteins. The three-dimensional structures of many proteins remain to be determined. Second, template-based modelling largely relies on the homology between target and template proteins. When the target and template proteins display a sequence similarity of >30%, the use of sequence alignment methods (e.g., BLAST [11] and SSEARCH [12]) can reveal their evolutionary relationships. However, this approach is not available for non-obvious relationships between targets and templates with a sequence identity of lower than 20%–30%. Third, template-based structure modeling is time consuming. This approach always requires homology detection by searching target proteins against a template database to detect distant evolutionary relationships.

To address the aforementioned problems, recent research efforts have focused on the development of template-free methods. Template-free methods seek to build models and accurately predict protein structures solely based on amino acid sequences rather than on known structural proteins as templates. Many machine learning algorithms have been recently used for that purpose; these algorithms include Hidden Markov Model (HMM), genetic algorithm, Artificial Neural Network, Support Vector Machines (SVMs), and ensemble classifiers. A key underlying assumption in employing machine learning-based methods for protein fold recognition is that the number of protein fold classes is limited [13]. Machine learning aims to build a prediction model by learning the differences between different protein fold categories and use the learned model to automatically assign a query protein to a specific protein fold class. This approach is thus more efficient for large-scale predictions and can examine a large number of promising candidates for further experimental validation. This review focuses mainly on the recent progress in machine learning-based methods for protein fold recognition. This review is organized as follows: First, we introduce the public databases usually used in protein fold recognition research. Second, we describe the framework and flowchart of machine learning-based recognition methods. Third, we summarize some recent representative machine learning-based methods for protein fold recognition. Finally, we evaluate and compare the recognition performance of existing methods used in the last 10 years on a benchmark dataset.

## 2. Databases

**Figure 1:**
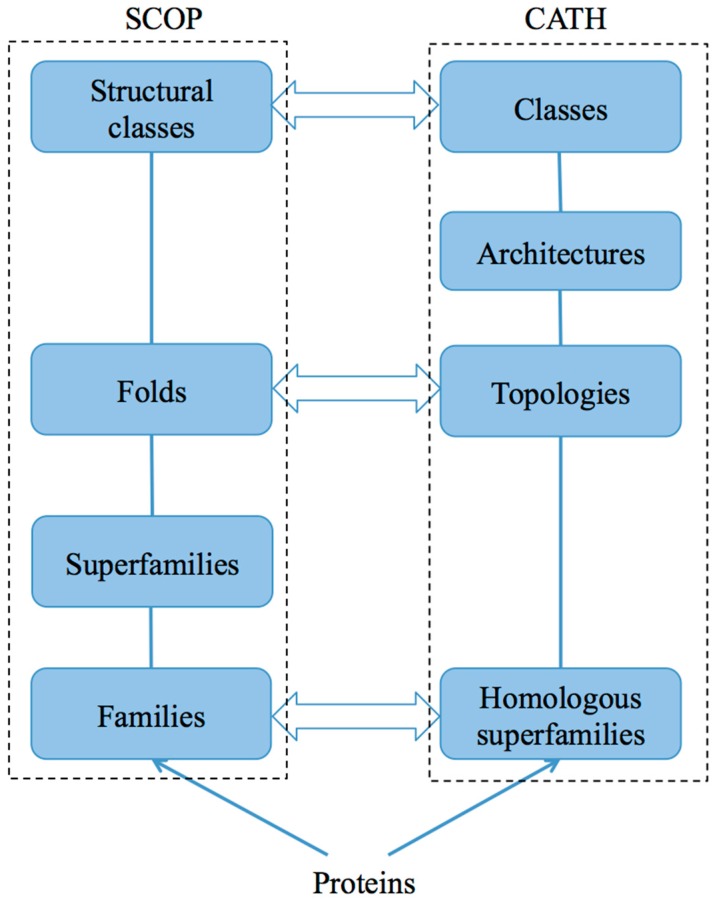
Architectures of two protein databases: SCOP and ACTH.

Multiple database sources are often used in protein structure research. These databases include PDB [14]; Universal Protein Resource [15]; Database of Secondary Structure of Protein (DSSP) [16]; Structural Classification of Proteins (SCOP) [17]; SCOP2 (a successor of SCOP) [18]; and Class, Architecture, Topology, Homology (CATH) [19] (Table 1). Among these databases, SCOP and CATH have become valuable resources in protein fold recognition research. Figure 1 shows the architectures of these databases. These databases are detailed below.

### 2.1. SCOP and SCOP2

SCOP, proposed by Murzin et al. [14], is a hierarchical protein classification database that aims to organize structurally characterized proteins based on their structural and evolutionary relationships. Proteins in SCOP are categorized into four hierarchical levels: family, superfamily, protein fold, and structural class. At the family level, proteins are clustered into families based on one of two principles; the first principle is that proteins display more than 30% sequence identity, and the second is that the proteins with lower sequence identities share similar structure and functions. Families containing proteins with low sequence identities but with similar structural and functional features and sharing a common evolutionary origin are grouped into superfamilies. At the fold level, superfamilies and families are clustered into a fold if their proteins display the same secondary structures in the same arrangement with similar topological connections. At the structural class level, different folds are grouped into classes for the convenience of users. In SCOP, seven different structural classes are formed based on protein secondary structure contents: (1) all α; (2) all-β; (3) α and β; (4) α plus β; (5) multi-domain proteins; (6) membrane and cell surface proteins; and (7) small proteins.

Murzin et al. [14] have recently presented a successor of SCOP, called SCOP2, which is available at http://scop2.mrc-lmb.cam.ac.uk. Compared with SCOP, SCOP2 displays a more advanced framework for protein structure classification, wherein the best features of SCOP are retained and a novel approach for classification of protein structures is offered. In SCOP2, protein sequences and their structures are presented in a directed acyclic graph to form a network of many-to-many relationships.

### 2.2. CATH

Similar to SCOP, CATH is a hierarchical protein domain classification. In the CATH database, proteins and their structures are obtained from the PDB database. When proteins share a clear common evolutionary ancestor, they are clustered into a homologous superfamily (“H” level in CATH, Figure 1). When proteins in the same homologous superfamily display the same fold but do not obviously show evolutionary relationships, they are grouped into the same topology (“T” level). Proteins in the “T” level show similar secondary structural arrangements and are clustered into the same architecture (“A” level). For that end, the architectures are further grouped into structural classes (“C” level) according to secondary structure content.

## 3. Framework of Machine Learning-Based Methods

**Figure 2:**
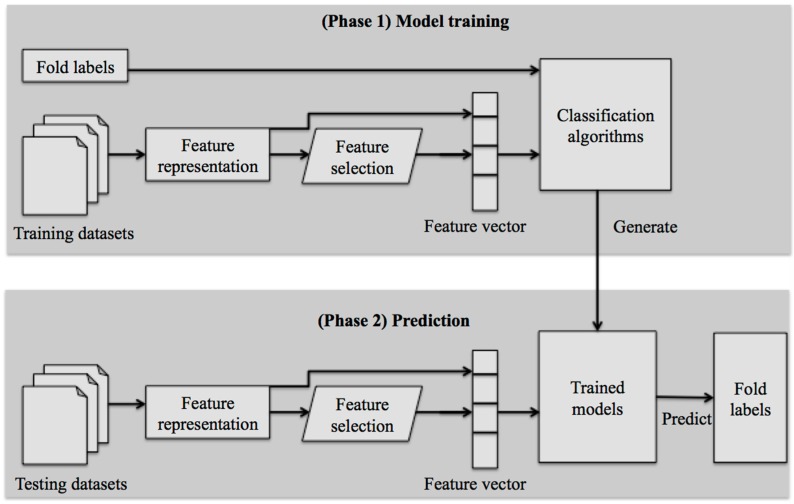
Framework of machine learning-based methods for protein fold recognition.

This section describes the mechanism of protein fold recognition by machine learning-based methods. The overall procedure in protein fold recognition by machine learning-based methods includes two phases (Figure 2): (1) model training; and (2) prediction.

In the first phase (model building), query protein sequences are first submitted into a pipeline of feature representation, in which sequences of different lengths are encoded with fixed-length feature vectors by feature descriptors. The commonly used feature descriptors include Amino Acid Composition (AAC), Pseudo AAC, Functional Domain (FunD), Position Specific Scoring Matrix (PSSM)-based descriptors, Secondary Structure-based descriptors, and Autocross-covariance (ACC) transformation. When the resulting feature representations display some irrelevant features or redundant features, an alternative step is usually performed to select the optimal feature subsets, which can yield the best performance, from the resulting feature representations. Subsequently, the feature vectors are fed into a pre-selected classification algorithm to train a prediction model. Typical classification algorithms often used in model building include SVM, Random Forest (RF), Naïve Bayes (NB), and Logistic Regression (LR). The first phase is completed in this step.

In the second phase (prediction), uncharacterized query proteins are first submitted into the same pipeline of feature representation as in the first phase. Note that if feature optimization of the generated feature representation is performed in the first phase, feature optimization should also be performed in the second phase; otherwise, the resulting feature vectors are fed into the trained prediction model, wherein the protein fold class to which the query proteins belong is predicted.

## 4. Recent Representative Methods for Protein Fold Recognition

Machine learning-based methods can be further categorized into two classes according to the learning algorithms used in prediction models: (1) single classifier-based methods; and (2) ensemble classifier-based methods. Single classifier-based methods use a single specific learning algorithm to build prediction models, whereas ensemble classifier-based methods use an ensemble of multiple, either similar or different, learning algorithms to build prediction models. This section introduces the recent single classifier-based methods and ensemble classifier-based methods used in protein fold recognition, as follows:

### 4.1. Single Classifier-Based Methods

Most of the current single classifier methods used in protein fold recognition are based on SVM classifier probably because SVM, being a well-known classification algorithm, has been highly efficient in several fields of bioinformatics, such as in protein remote homology detection, protein structural classification, and DNA-binding protein prediction. SVM-based protein fold recognition methods include Shamim’s method [20], Damoulas’ method [21], ACCFold_AC and ACCFold_ACC [22], TAXFOLD [23], and Alok Sharma’s method [24]. The main difference among these SVM-based methods is their feature representation algorithms. For instance, Shamim et al. [20] propose to use secondary structural state and solvent accessibility state frequencies of amino acids and amino acid pairs as feature vectors. Of these features, the secondary structural state frequencies are more effective than the two other features for fold class discrimination. Combining the secondary structural state frequencies with the two other feature types can further improve the accuracy of fold discrimination. Dong et al. [22] propose the ACCFold_AC and ACCFold_ACC methods for protein fold recognition. Based on the distant evolutionary relationships of protein sequences, their proposed feature algorithm can effectively capture the evolutionary information embedded in the form of position-specific score matrices and the sequence-order effect by utilizing ACC transformation. The TAXFOLD method, proposed by Yang et al. [23], proposes to use global and local sequential and structural features for protein fold classification. Given that an increasing number of features are proposed, simply fusing different types of feature spaces is probably not an informative means to further improve recognition accuracy. Thus, a classification method that can assess the contribution of these potentially heterogeneous object descriptors must be developed. For this reason, Damoulas et al. [21] propose a single multi-class kernel machine that informatively combines available feature groups. Apart from the SVM classifier, other single classifiers, such as RF (Random Forest) [25] and Hidden Markov Model [26], are used to construct a prediction engine in machine learning-based methods.

Chen et al. [25] recently propose an RF-based protein fold recognition method called PFP-RFSM. The framework of PFP-RFSM involves a comprehensive feature representation algorithm that can capture distinctive sequential and structural information from primary sequences and predicted structures, respectively. This feature representation algorithm generates features from seven perspectives, namely: amino acid composition, secondary structure contents, predicted relative solvent accessibility, predicted dihedral angles, PSSM matrix, nearest neighbor sequences, and sequence motifs. Features based on sequence motifs are utilized in protein fold recognition for the first time. Moreover, the PFP-RFSM method is the first to use the RF classifier as its prediction engine. As reported in [25], RF classifier is superior over the other commonly used classifiers, such as SVM, NB, and LR. In terms of overall performance, RF outperforms most of the existing methods, especially some of the ensemble-classifier methods (e.g., the well-known PFP-FunDSeqE method).

Lampros et al. [26] propose a novel optimization method for protein fold classification; the prediction model of this method is constructed based on a Markov chain trained with primary structure of proteins and on a reduced state-space HMM, which is an effective means of classifying proteins in fold categories with low computational cost. The proposed Markov chain requires only a primary sequence for training, and it is trained using a likelihood maximization algorithm. This method has proven to be effective in protein fold categorization [26].

### 4.2. Ensemble Classifier-Based Methods

**Figure 3:**
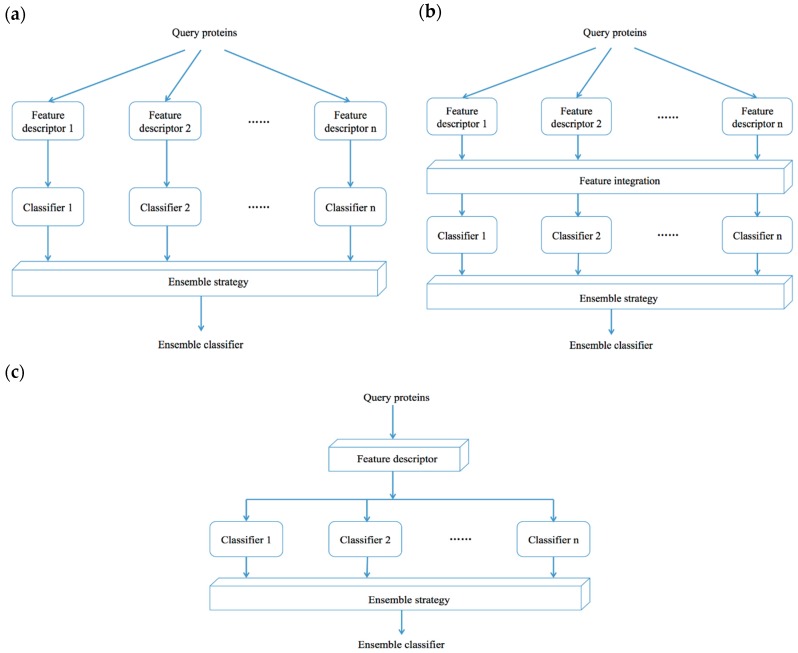
Construction types of ensemble-classifier models.

Most of the recently developed methods for protein fold recognition are based on ensemble classifier models. Figure 3 shows the three general types of ensemble classifier models. For given *n* different single basic classifiers, the first type of ensemble classifier-based methods use one specific feature descriptor to encode query proteins with feature representations (Figure 3a); the feature representations are trained with each single basic classifier to create *n* single classifier models, and then all of the *n* trained single classifier models are combined with ensemble strategies to generate an ensemble classifier-based model. For a given *n* different single basic classifiers and *n* different feature descriptors, the second type of ensemble classifier-based methods use *n* feature descriptors to encode query proteins with *n* different feature representations (Figure 3b); the *n* feature representations are sequentially combined as one to train the *n* single basic classifiers, and then all of the *n* trained single classifier models are combined with ensemble strategies to generate an ensemble classifier-based model. For a given specific classifier and *n* different feature descriptors, the third type of ensemble classifier-based methods use *n* feature descriptors to encode query proteins with *n* different feature representations (Figure 3c); the *n* feature representations are respectively trained with a specific single classifier to construct *n* single classifier-based models, and then all of the *n* trained single classifier models are combined with ensemble strategies to generate an ensemble classifier-based model. This section highlights some representative ensemble methods used in protein fold recognition.

One well-known ensemble classifier method is PFP-FunDSeqE proposed by Shen and Chou [27]. In PFP-FunDSeqE, a novel feature extraction approach is proposed to explore the functional domain information and sequential evolution information. This approach generates 17,402 FunD features and 220 Pseudo PSSM features. The two feature groups are separately fed into an optimized evidence-theoretic K-nearest neighbor (OET-KNN) classifier to build prediction models. Accordingly, the two optimized OET-KNN models are fused to generate an ensemble classifier prediction model.

Wei et al. [13] also develop an ensemble method called PFPA. In the PFPA method, the authors design a novel feature representation algorithm considering the sequential evolutionary information and structural information. The sequential evolutionary information is derived from PSI-BLAST [28] and profiles which are generated by searching query proteins against a non-redundancy database. On the basis of the PSI-BLAST profiles, the authors compute 20 PSSM features and 420 amino acid compositional features from consensus sequences, which contain rich evolutionary information. The structural information is derived from PSI-PRED [29] profiles. To sufficiently explore the structural information, the authors calculate 27 local and 6 global secondary structure features from PSI-PRED profiles. Generally, all of the sequential and structural features are integrated to construct comprehensive feature representations of query proteins. For prediction engine construction, they build an ensemble classifier model, which fuses five basic classifier models (RF [30], NB [31], Bayes Net [32], LibSVM [33], and SMO (Sequential Minimal Optimization) [34]) with an average probability strategy. Importantly, an online webserver that implements the PFPA method is developed and freely available at http://server.malab.cn/PFPA/index.html. This method is useful to researchers in this field.

Moreover, Chen et al. [35] recently proposed a recognition method called ProFold. In ProFold, information on protein tertiary structures is first considered in its feature extraction framework in addition to other commonly used features, such as global features of amino acid sequence, PSSM features, functional domain features, and physiochemical features. The tertiary structure features are used to compute eight types of secondary structure states from PDB files by using DSSP. Additionally, ProFold proposes a novel strategy to construct an ensemble classifier. The authors first select 10 widely used basic classifiers, such as Logistic model tree [36], RF [30], LibSVM [33], Simple Logistic [36], Rotation Forest [37,38], SMO [34], NB [31], Random Tree [30], Functional tree [39], and Simple Cart [40]. Subsequently, different types of feature representations are trained using these 10 basic classifiers. For each feature type, the model with the highest accuracy is chosen, generating four single classifier models for the four feature types. The four models are DSSP model, AAsCPP model, PSSM model, and FunD model. The average probability strategy is used to fuse the four single classifier models, similar to that in the PFPA method.

## 5. Comparisons with Different Methods on Benchmark Dataset

To examine the effectiveness of existing machine learning-based methods in the literature for protein fold recognition, an intuitive comparison is to perform the methods on a public benchmark dataset. Here, a public and stringent dataset, proposed by Ding and Dubchak [41], is employed as a benchmark dataset for performance comparison of the existing methods. This dataset, referred as to DD, has been widely used in several studies [22,23,27,41,42,43,44,45,46,47,48,49,50]. The DD dataset is comprised of a training dataset and a testing dataset, both of which cover 27 protein fold classes in the SCOP database. The training dataset contains 311 protein sequences with ≤40% residue identity, while the testing dataset contains 383 protein sequences with ≤35% residue identity. Importantly, the sequences in the training dataset have residue identity ≤35% with that in testing dataset, thus ensuring to provide unbiased performance evaluation. The sequence distribution of each of the 27-fold classes can be seen in Table 2.

As the benchmark dataset determined, the next thing is to determine the methods for performance comparison. To provide a comprehensive comparison, we evaluated and compared the 20 representative methods published in the past 10 years (from 2006 to present) on the DD dataset. The compared 20 methods are first modeled by the training dataset of the DD dataset, and then they are tested on the testing dataset of the DD dataset. The prediction results are presented in Table 3. As shown in Table 3, we observe the following two experimental results. First, the recent ProFold exhibits the best performance among other existing methods. The overall accuracy of ProFold is 76.2%, which is 2.6%–15.7% higher than that of other methods. This demonstrates that the ProFold has great power to distinguish the 27-fold classes in the DD dataset. The significant performance improvement of ProFold contributes to the first use of the DSSP feature in the field of protein fold recognition. Their research results indicate that integrating the DSSP features into feature representations remarkably enhanced the overall accuracy from 71.2% to 76.2% [35]. This provides an alternative way to further improve predictive performance by integrating some unexplored but informative features. Second, of the 20 methods, 14 methods are based on an ensemble classifier, while 6 methods are based on a single classifier. In particular, we observe that there are 9 out of 20 methods that obtain an overall accuracy of >70%, which are PFP-FunDSeqE (70.5%), TAXFOLD (71.5%), Marfold (71.7%), Kavousi et al. (73.1%), PFPA (73.6%), Feng and Hu (70.2%), Feng et al. (70.8%), and ProFold (76.2%), respectively. Of the nine methods, only TAXFOLD is based on single classifier while the other methods are based on ensemble classifier. This indicates that ensemble classifiers are more effective than single classifiers for protein fold recognition. On the other hand, this result also explains why more recent research efforts are focused on the development of ensemble-classifier-based predictors.

## 6. Conclusions and Perspectives

We have systematically reviewed the recent progress in machine learning-based protein fold recognition methods. Compared with the traditional experimental methods, machine learning-based methods present three advantages. First, they demonstrate accurate, robust, and reliable performance. Second, they can be applied in large-scale protein fold recognition; this application is extremely important in the post-genomic era, wherein numerous proteins remain to be structurally characterized. Third, they can effectively address the intrinsic limitations of experimental methods, that is, their being time consuming and expensive. In the past decades, remarkable progress has been made in computational protein fold recognition. However, several challenges remain to be addressed.

First, the benchmark dataset (e.g., DD dataset) used to evaluate the performance of predictors actually suffers some limitations. For instance, the DD dataset is imbalanced. Table 2 shows that the ratio of the smallest class (“EF hand-like”) against the largest class (“immunoglobulin-like β-sandwich”) is roughly 1:4. Moreover, the sample size for each fold class is small. Only 383 training sequences belong to 27-fold classes. The largest fold class contains 30 training samples, whereas the smallest fold class contains 6 training samples. Generally, the prediction model generated based on such an imbalance and small dataset is easily overfitting.

Second, most of the existing methods, especially for those with online webservers, can only provide for the populated 27-fold class prediction. Although the sequences of the 27-fold classes cover the majority of the sequences in SCOP database, approximately 1800 protein fold classes actually exist in SCOP. Thus, developing adaptive multi-class protein fold predictors is desirable given that an increasing number of protein fold classes are being discovered.

Third, constructing informative and effective prediction engines remains a great challenge. Well-established ensemble classifiers have demonstrated their classification power in protein fold recognition. The use of deep learning algorithms for classification tasks has been a recent research hotspot in the machine learning field. Deep learning networks have been successfully applied in protein fold recognition [57]. Combining deep learning networks with well-established ensemble classifiers is probably an alternative means to improve the efficiency of protein fold recognition.

In general, machine learning-based methods can be successfully applied in protein fold recognition. In the future, machine learning methods will be extensively applied in other similar but unexplored fields, such as disease-causing amino acid change prediction [58,59,60], protein-protein binding site or interaction prediction [61,62,63], and DNA-protein binding site or interaction prediction [64,65,66].

## Figures and Tables

**Table 1:** Summary of database sources of protein structure classification.

Database Sources	Websites	References
PDB	http://www.rcsb.org/pdb/	[14]
UniProt	http://www.uniprot.org/	[15]
DSSP	http://swift.cmbi.ru.nl/gv/dssp/	[16]
SCOP	http://scop.mrc-lmb.cam.ac.uk/	[17]
SCOP2	http://scop2.mrc-lmb.cam.ac.uk/	[18]
CATH	http://www.cathdb.info/	[19]

**Table 2:** Sequence distribution of the 27-fold classes in the DD dataset.

Index	Fold Identifier	Fold Name	S_Train_	S_Test_	Total
1	a.1	Globin-like	13	6	19
2	a.3	Cytochrome c	7	9	16
3	a.4	DNA/RNA-binding 3-helical bundle	12	30	32
4	a.24	4-Helical up-and-down bundle	7	8	15
5	a.26	4-Helical cytokines	9	9	18
6	a.39	EF hand-like	6	9	15
7	b.1	Immunoglobulin-like β-sandwich	30	44	74
8	b.6	Cupredoxin-like	9	12	21
9	b.121	Nucleoplasmin-like/VP	16	13	29
10	b.29	ConA-like lectins/glucanases	7	6	13
11	b.34	SH3-like barrel	8	8	16
12	b.40	OB-Fold	13	19	32
13	b.42	β-Trefoil	8	4	12
14	b.47	Trypsin-like serine proteases	9	4	13
15	b.60	Lipocalins	9	7	16
16	c.1	TIM β/α-barrel	29	48	77
17	c.2	FAD/NAD(P)-binding domain	11	12	23
18	c.3	Flavodoxin-like	11	13	24
19	c.23	NAD(P)-binding Rossmann	13	27	40
20	c.37	P-loop containing NTH	10	12	22
21	c.47	Thioredoxin-fold	9	8	17
22	c.55	Ribonuclease H-like motif	10	12	22
23	c.69	α/β-Hydrolases	11	7	18
24	c.93	Periplasmic binding protein-like	11	4	15
25	d.15	β-Grasp (ubiquitin-like)	7	8	15
26	d.58	Ferredoxin-like	13	27	40
27	g.3	Knottins (small inhibitors, toxins, lectins)	13	27	40
Total			311	383	694

Note that S_Train_ denotes the training dataset, and S_Test_ denotes the testing dataset.

**Table 3:** Performance of representative machine learning-based methods in the literature on the DD dataset.

Index	Methods	Classifier Type	References	Overall Accuracy (%)
1	Nanni et al. (2006)	Ensemble	[49]	61.1
2	PFP-Pred (2006)	Ensemble	[50]	62.1
3	Shamim et al. (2007)	Single (SVM)	[20]	60.5
4	PFRES (2007)	Ensemble	[42]	68.4
5	Damoulas et al. (2008)	Single (SVM)	[21]	68.1
6	ALHK (2008)	Ensemble	[51]	61.8
7	GAOEC (2008)	Ensemble	[52]	64.7
8	PFP-FunDSeqE (2009)	Ensemble	[27]	70.5
9	ACCFold_AC (2009)	Single (SVM)	[22]	65.3
10	ACCFold_ACC (2009)	Single (SVM)	[22]	66.6
11	Ghanty et al. (2009)	Ensemble	[47]	68.6
12	TAXFOLD (2011)	Single (SVM)	[23]	71.5
13	Alok Sharma et al. (2012)	Single (SVM)	[24]	69.5
14	Marfold (2012)	Ensemble	[53]	71.7
15	Kavousi et al. (2012)	Ensemble	[54]	73.1
16	PFP-RFSM (2013)	Single (RF)	[25]	73.7
17	Feng and Hu (2014)	Ensemble	[55]	70.2
18	PFPA (2015)	Ensemble	[13]	73.6
19	Feng et al. (2016)	Ensemble	[56]	70.8
20	ProFold (2016)	Ensemble	[35]	76.2
